# The Quantitative Genetics of Phenotypic Robustness

**DOI:** 10.1371/journal.pone.0008635

**Published:** 2010-01-08

**Authors:** Hunter B. Fraser, Eric E. Schadt

**Affiliations:** 1 Department of Biology, Stanford University, Stanford, California, United States of America; 2 Pacific Biosciences, Menlo Park, California, United States of America; 3 Department of Medical Genetics, University of Washington, Seattle, Washington, United States of America; Georgia Institute of Technology, United States of America

## Abstract

Phenotypic robustness, or canalization, has been extensively investigated both experimentally and theoretically. However, it remains unknown to what extent robustness varies between individuals, and whether factors buffering environmental variation also buffer genetic variation. Here we introduce a quantitative genetic approach to these issues, and apply this approach to data from three species. In mice, we find suggestive evidence that for hundreds of gene expression traits, robustness is polymorphic and can be genetically mapped to discrete genomic loci. Moreover, we find that the polymorphisms buffering genetic variation are distinct from those buffering environmental variation. In fact, these two classes have quite distinct mechanistic bases: environmental buffers of gene expression are predominantly sex-specific and trans-acting, whereas genetic buffers are not sex-specific and often cis-acting. Data from studies of morphological and life-history traits in plants and yeast support the distinction between polymorphisms buffering genetic and environmental variation, and further suggest that loci buffering different types of environmental variation do overlap with one another. These preliminary results suggest that naturally occurring polymorphisms affecting phenotypic robustness could be abundant, and that these polymorphisms may generally buffer either genetic or environmental variation, but not both.

## Introduction

Variation is ubiquitous in biology. The sources of non-random phenotypic variation within species can be genetic, caused by alleles segregating within a population, or environmental, caused by the fluctuating external conditions all organisms face. Waddington, who introduced the concept of canalization over 65 years ago [1], observed that “wild-type” individuals are often quite insensitive to both genetic and environmental variation. In other words, they are robust.

Genetic robustness (GR) is the insensitivity of a trait to variation in the genome. Thus when multiple individuals experience the same genetic perturbation(s), the one with less change in the trait's value has higher GR. Such perturbations can be artificially introduced via mutagenesis in a laboratory, or can be naturally occurring polymorphisms within a species (because only the latter can impact the evolution of species in the wild, we concentrate on natural variation in this study).

Analogously, environmental robustness (ER) is the insensitivity of a trait to variation in the environment. Environmental variation can either be obvious (such as large fluctuations in temperature) or subtle (such as the inevitable micro-environmental variation always present even in “controlled” experiments), but in either case the concept of ER is the same.

Despite the ubiquity of phenotypic robustness, we still lack even a basic understanding of how robustness to different perturbations comes about. In particular, one fundamental but largely unexplored question is to what extent robustness is polymorphic within species. This has important implications for our understanding of the evolution of robustness, since polymorphism is necessary for evolution (via either natural selection or random drift) to occur. Another major unresolved question is whether the factors buffering genetic variation are the same as those buffering environmental variation. Evidence from the numerous studies addressing this question falls into three general categories: theoretical evidence, indirect empirical evidence, or direct empirical evidence.

Some theoretical studies [2]–[3] have concluded that GR may only be selected for under very restricted conditions, whereas selection for ER shares no such restrictions. For this reason these studies suggest that GR may exist only because factors buffering environmental variation exert a similar (though selectively neutral) canalizing effect on genetic variation. However, such studies do not demonstrate whether environmental buffers are actually capable of buffering genetic variation. A different approach was taken in a pioneering theoretical study [4] that used computational modeling of RNA secondary structure to demonstrate an association between ER and GR; but whether this finding also applies to living organisms has not been established.

Indirect empirical studies usually show an association between GR and ER, either across species or across phenotypes. For example, in a study of five traits in *Drosophila melanogaster*, the traits with highest GR also had the highest ER [5]. Likewise among five strains of an RNA virus, GR (for plaque size) correlated with ER [6]. Indirect evidence is not conclusive, however, because alternative explanations (other than GR and ER being caused by the same factors) are quite plausible. For example, in the RNA virus study [6], the strains with greatest robustness were also the ones that had accumulated the most deleterious mutations, so it is possible that factors responsible for the changes in GR and ER were independently mutated in these most-mutated strains. In the *Drosophila* study [5], different traits had different mutational variances, confounding any comparison of robustness between traits [7]. Finally, GR and ER can also show a negative association [8], or no association [9]–[10].

Direct empirical investigations have been rare, despite having the potential to provide the most convincing answer to this question. Hsp90, the most well-studied buffer of genetic variation, has also been shown to buffer micro-environmental variation in *Arabidopsis thaliana* [11]–[12] and *Drosophila* [13]. The most important limitation of these studies for answering questions about phenotypic robustness in general is that it is not clear if the results from Hsp90 will apply to the hundreds or thousands of other buffering factors present in living systems [14] as well.

Quantitative genetics offers a promising approach to disentangling the genetic and environmental components of phenotypic robustness. In particular if the robustness of some trait is polymorphic within a species, and if genomic regions that contribute to polymorphic GR or ER could be genetically mapped to quantitative trait loci (QTL), then a comparison of the regions contributing to each type of buffering would indicate whether the same factors (or at least the same regions of the genome) contribute to each type of buffering.

Loci influencing polymorphic ER of morphological traits have been genetically mapped in *Saccharomyces cerevisiae* [15], *Arabidopsis* [12], [16], and *Drosophila* [17]. QTL affecting the related phenomenon of developmental stability (typically measured by fluctuating asymmetry–variation in a trait that is repeated at least twice in each individual, such as the size of teeth on the left vs. right sides of a mouse) have also been mapped [18]–[19]. In contrast to ER, no loci influencing GR have yet been mapped (see Materials and Methods).

We have developed a framework for the genetic mapping of alleles that influence the buffering of environmental and genetic variation. By applying this framework to genome-wide gene expression data, we are able to explore ER and GR in the context of thousands of traits simultaneously, providing the means to empirically characterize general properties of ER and GR, and how they relate to one another. Using this methodology, we present an analysis of the genetic architecture of phenotypic robustness.

## Results

### A Method for Genetic Mapping of Phenotypic Robustness

The GR for any group of strains (composed of genetically identical individuals) can be measured as a trait's between-strain variation; this GR can then be compared with that of another group (containing comparable natural genetic variation). If strains within a species differ in their GR, then their GR is polymorphic (note that a strain could have higher GR for one trait but lower GR for another). Polymorphic GR is a form of epistatic gene-gene interaction that uncovers cryptic genetic variation: in the strains with higher GR, genetic variation is (by definition) suppressed, resulting in a constant trait value even in the presence of a varying genetic background. If a difference in GR between two groups of strains is caused by a single polymorphic factor, where one allele is a more effective buffer than the other, then the polymorphism is epistatically interacting with at least one (and perhaps many) other polymorphism(s). The observed difference in GR is the result of this interaction. Not all epistatic interactions affect GR, however. For example, if the direction of effect of one allele depends on the genotype at a second locus, but the trait variance is not affected by the genotype at the second locus, then this is not a GR QTL. We note that while some factors (e.g. Hsp90) buffer genetic variation at many loci, factors that buffer only one or a few polymorphisms fit equally well into the definition of GR.

The situation is similar for polymorphic ER. A trait's ER can differ between two groups of strains due to a polymorphic factor where one allele buffers environmental variation more effectively than the other. In this case, it is a gene-environment interaction, meaning the effect of environmental variation depends on the genotype at the buffering locus. In such a context, ER can be quantified by the within-strain variation of a trait.

**Figure 1:**
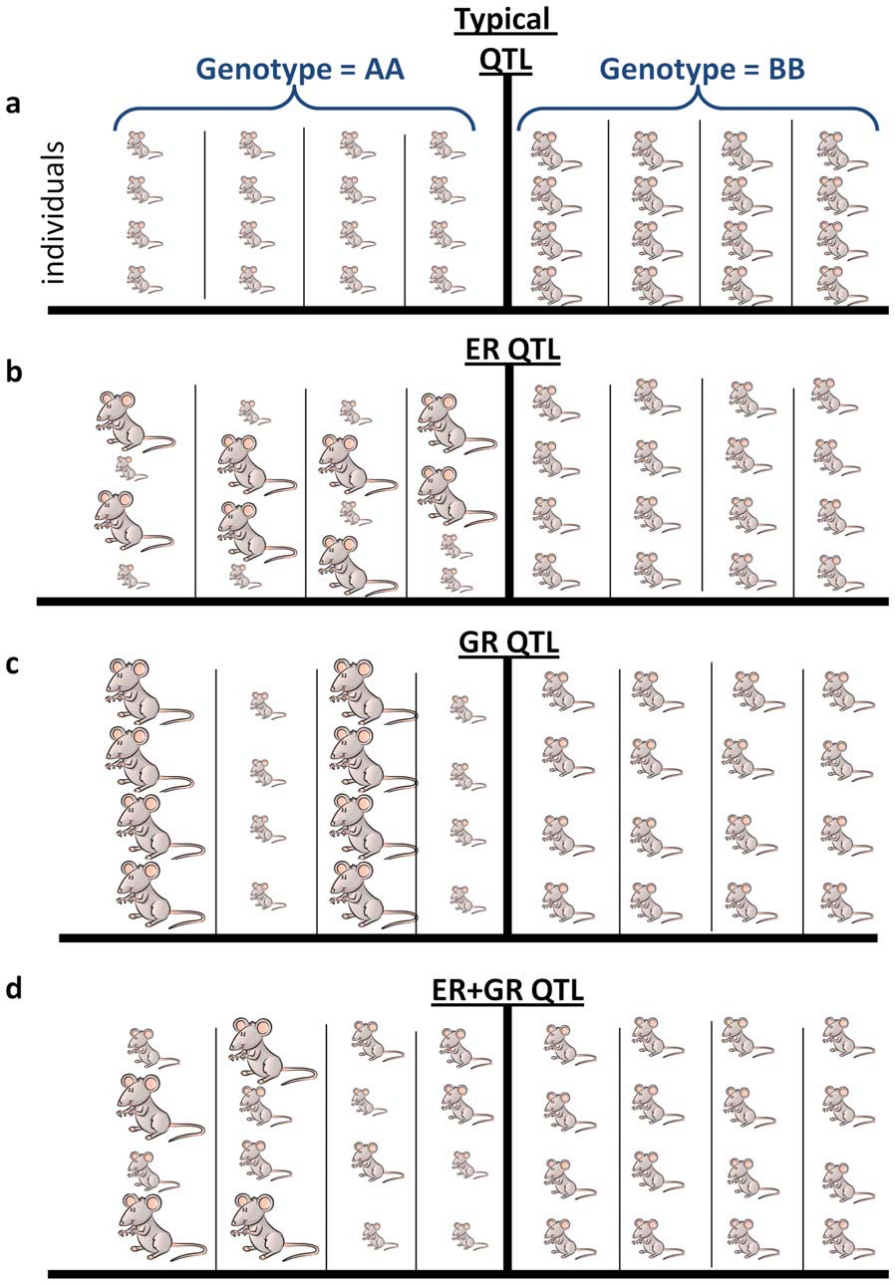
Types of QTL discussed. For each QTL type, four genetically identical mice from each of eight inbred strains (columns) are shown. Half of the strains have genotype AA at a particular genetic marker (left panes), and half have genotype BB (right panes). **a.** A “typical” QTL that affects the trait mean: all individuals with genotype BB are larger than any individuals of genotype AA. Thus a polymorphism linked to the genetic marker affects the trait's mean value. **b.** An environmental robustness (ER) QTL: The mean trait value is identical for each strain, but there is greater within-strain variance for strains with genotype AA. Thus a polymorphism linked to the genetic marker affects the trait's sensitivity to the environment (a gene-environment interaction). **c.** A genetic robustness (GR) QTL: The mean trait value is identical for each genotype, but not for each strain; there is greater between-strain (but not within-strain) variance for strains with genotype AA. Thus a polymorphism linked to the genetic marker affects the trait's sensitivity to the genetic background (an epistatic gene-gene interaction). **d.** A combined ER+GR QTL: Again the mean trait value is identical for each genotype, but there is both greater between-strain and within-strain variance for strains with genotype AA. Thus a polymorphism linked to the genetic marker affects the trait's sensitivity to both the genetic background and the environment.

It is important to understand how QTL for ER and GR differ from more “typical” QTL. A typical QTL is one where the mean of a trait is significantly associated with the genotype at some genetic marker(s), indicating that some polymorphism(s) linked to the marker(s) (or the marker itself) affect the associated trait. Fig. 1a illustrates a QTL affecting the mean size of inbred strains of mice: four individuals from each of eight strains are shown, and all 16 individuals with the AA QTL genotype (left pane) are smaller than any of the 16 BB individuals (right pane). Also note the lack of within-strain variation: all individuals within any single strain (columns) have equal sizes. This is in contrast to an ER-QTL (Fig. 1b), where within each AA strain half of the individuals are smaller than the BB mean and half are larger, though the mean size is no different between the genotypes. Because individuals within any inbred strain are essentially genetically identical, the increased within-strain variances in AA strains reflect decreased ER (stochastic differences between individual cells may also contribute to the within-strain variance when the phenotype in question is at the single-cell level). In this example, we can conclude that the polymorphic ER is likely due to a polymorphism linked to the marker whose genotype (AA/BB) is shown. As mentioned above, this type of ER QTL mapping has been applied previously [12], [15]–[17].

Alleles affecting GR can be mapped in an analogous way. Instead of differences in within-strain variation (Fig. 1b), the signature of polymorphic GR is a difference in the between-strain variation (Fig. 1c). Using the median phenotype value of each inbred strain will substantially reduce micro-environmental effects (assuming no systematic differences in the environment for the different strains); any difference in the dispersion of medians within one genotype group (AA) versus the other (BB) then indicates polymorphic GR. Despite the existence of an extensive literature concerning ER and GR [1]–[17] and studies that have genetically mapped ER QTL [12], [15]–[17], no previous studies have mapped GR QTL, and no study has systematically explored GR and ER in the context of thousands of molecular traits. Finally, an allele that affects both GR and ER will cause differences in both within- and between-strain variation (Fig. 1d), again with no difference in the trait mean between the two genotype classes.

Any standard QTL mapping techniques can be applied to mapping ER alleles, simply substituting within-strain trait variances for the (typically used) within-strain trait means. However, to map GR alleles the variation between entire groups of strains (segregated by the genotype at some marker, as in Fig. 1c) must be compared. Many statistical tests exist for contrasting the variation in multiple groups; we chose to use a variant of the Fligner-Killeen test (see Materials and Methods), which is a test of dispersion that has been shown to be more robust and powerful than scores of other such tests [20]–[21], but has not been applied to genetic mapping. Because it is a non-parametric rank-based test, it does not require any particular distribution of data points, and is robust to outliers. To ensure that only effects on trait variability were considered, in all analyses we discarded any trait/marker pairs with even marginally (nominal p<0.01) significant association between marker genotype and trait median (see Materials and Methods).

Our approach is attractive in several respects. First, it is unbiased in the sense that it can be applied to any quantitative trait and any genetic polymorphism, much like traditional genetic mapping methods; in fact it can even be applied to previously published data sets, where appropriate replication of phenotype measurements in genetically identical individuals exists. Second, it uses the same individuals/phenotypes/genetic markers to map both GR and ER, allowing for straightforward comparison between the results of each. And third, it is computationally efficient, allowing millions of trait/marker pairs to be analyzed in a short time frame.

### Mapping Genetic Robustness in Mouse

We chose to use gene expression levels as our quantitative traits, because they are numerous–allowing thousands of traits to be studied simultaneously–and have been shown to be amenable to quantitative genetic analysis [22]. To this end we generated genome-wide gene expression measurements from the livers of ∼20 individuals from each of 19 diverse inbred mouse lines (see Materials and Methods)–a total of 370 mice (182 females and 188 males). These inbred lines have previously been genotyped at ∼157,000 single-nucleotide polymorphisms [23], providing a dense set of genetic markers for testing.

**Figure 2:**
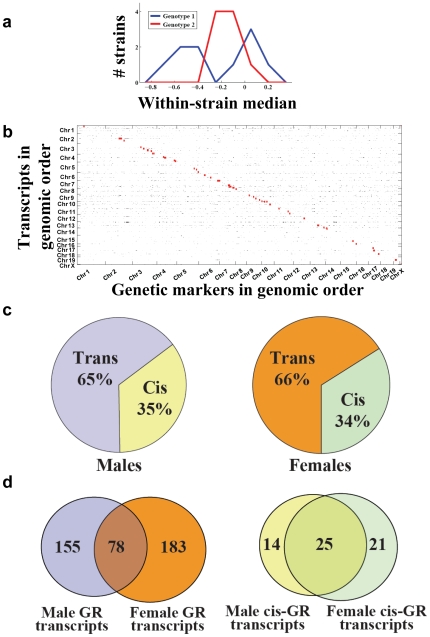
Genetic robustness QTLs in mouse. **a.** An example of a GR QTL in the mouse gene expression data set. A histogram of expression levels from mice of one genotype (red) form a tight unimodal distribution, while those of the other genotype (blue) form a much wider bimodal distribution. The median values for each genotype are required not to be significantly different. **b.** GR QTLs in males. Transcripts are arranged in the genomic order of their genes along the Y-axis and genetic markers are in genomic order along the X-axis. Small black dots located at the intersection of a particular row and column indicate trans-acting hits between the trait/marker combination represented by that row/column; larger red dots indicate cis-acting hits. **c.** Left pane: the estimated fraction of true-positive male GR hits that is cis-acting. Right pane: the same as the left pane, for females. **d.** Left pane: the overlap between male and female GR QTLs. Right pane: the overlap between male and female cis-acting GR QTLs.

Applying our algorithm for mapping of GR loci (Fig. 1c) to the most informative microarray probes/markers (see Materials and Methods), we identified hundreds of gene expression traits at the maximum possible significance level for this data set (p<0.0001). An example of such a maximally significant hit is shown in Fig. 2a: one genotype group (nine strains) forms a tight cluster of median expression levels, while the other genotype group (ten strains) is split into a bimodal distribution straddling the tightly clustered group (Specifically, every expression level from the bimodal group is further from that group's median value than any expression level from the tightly clustered group is from that group's median, and the two medians are not significantly different from one another. As long as these two criteria are met, the actual values of the data do not affect the significance of our rank-based metric.). Because statistical power is limited with only 19 strains, our results should be interpreted as a coarse-grained view of robustness QTL.

Among males, 233 gene expression traits mapped at this maximal significance to an average of 20.2 non-redundant genetic markers each (Fig. 2b); in females, 261 traits mapped to 21.2 markers each (Fig. S1a) (finding ∼20 markers per trait was not surprising, considering the strong correlations between many marker pairs, usually those in close genomic proximity). To assess whether this could occur by chance, we permuted the data set and ran our algorithm on the randomized data (see Materials and Methods). Repeating this over ten-thousand times, we never observed as many associations as in the real data (p<0.0001 for both males and females), confirming that GR was polymorphic and genetically mappable for many transcripts. Tests of population stratification, batch effects, SNPs disrupting probe hybridization, and systematically inflated p-values all showed the lack of any confounding factors (see Materials and Methods).

Using gene expression levels as traits has an advantage over most other phenotypes: if the gene encoding the transcript in question is located close in the genome to a marker that is a significant hit for that trait, then it can be inferred to most likely be a cis-acting polymorphism. If instead the marker is far away from the gene or on a different chromosome, then it is likely trans-acting [22]. We classified an expression trait as likely having a cis-acting basis (a “cis hit”) if its GR mapped to at least one marker within 5 mb of the gene itself.

The number of cis hits we observed was greater than expected by chance. In males, 39 GR QTL were cis-acting (Fig. 2b red points; p<0.0001), and in females, 46 were in cis (Fig. S1a, red points; p<0.0001). We estimate that in males, 35% of all true-positive hits are cis-acting; in females, this figure is 34% (Fig. 2c) (see Materials and Methods). Therefore in both sexes, cis hits make up over a third of the loci influencing GR.

We found no evidence for any sex-specificity of GR QTL (see Materials and Methods); therefore the overlap between the male and female lists is reflective of our test's statistical power. This overlap consisted of 78 transcripts (Fig. 2d and Fig. S1b), indicating reasonably high power of our test. The male/female overlap among the cis-acting subset of hits was higher still, with 25 overlapping transcripts (Fig. 2d).

We did not find any GR QTL hotspots (loci where the GR of many transcripts maps), as can be seen from the lack of vertical “stripes” of points in Fig. 2b and Fig. S1: most markers were associated with only one of the 78 transcripts, and the maximum number of transcripts mapping to a marker was ten. Thus polymorphisms affecting GR appear each to influence only a small number of traits. Furthermore, the transcripts affected by GR QTL did not show any bias in functional annotation, suggesting that polymorphic GR is not limited to any particular annotated class of genes.

### Mapping Environmental Robustness in Mouse

**Figure 3:**
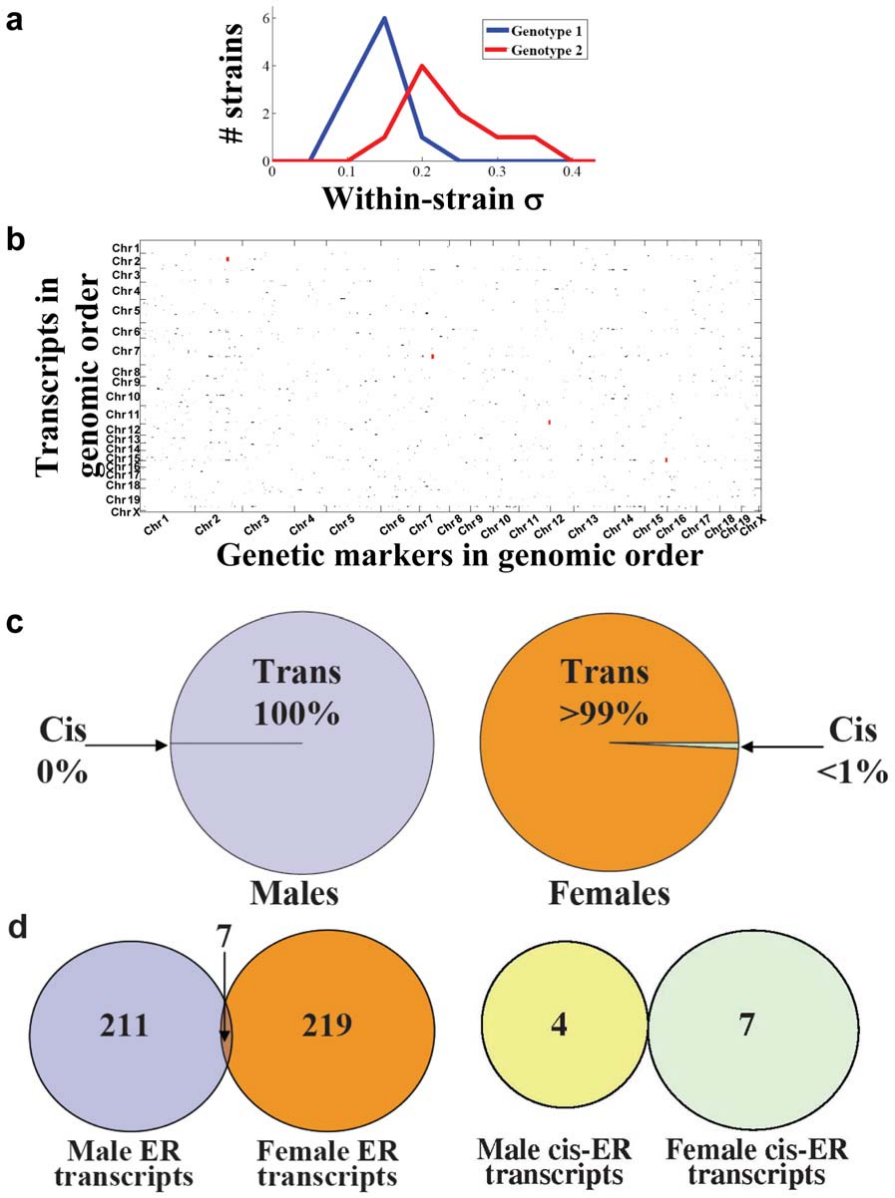
Environmental robustness QTLs in mouse. **a.** Example of an ER QTL in the mouse gene expression set. A histogram of the within-strain expression level standard deviations (σ), which are significantly greater for one genotype compared to the other. The median values for each genotype are required not to be significantly different. **b.** ER QTLs in males. Transcripts are arranged in the genomic order of their genes along the Y-axis and genetic markers are in genomic order along the X-axis. Small black dots located at the intersection of a particular row and column indicate trans-acting hits between the trait/marker combination represented by that row/column; larger red dots indicate cis-acting hits. **c.** Left pane: the estimated fraction of true-positive male ER hits that is cis-acting. Right pane: the same as the left pane, for females. **d.** Left pane: the overlap between male and female ER QTLs. Right pane: the overlap between male and female cis-acting ER QTLs.

We employed the same mouse gene expression data set described above to identify loci influencing ER, using the approach illustrated in Fig. 1b. A within-strain standard deviation was calculated for each trait/strain combination, which was analyzed using an additive model (see Materials and Methods). We found 211 transcripts mapping to an average of 7.6 markers each in males (p = 0.005; Fig. 3b), and 219 transcripts each mapping to 7.6 markers in females (p = 0.006; Fig. S2a). We did not detect any confounding factors affecting our ER QTL mapping (see Materials and Methods).

In contrast to GR QTL, for ER QTL we found only seven cis hits in females, and four in males (Fig. 3b red points and Fig. 3c). These are almost exactly what would be expected by chance in lists of these sizes (∼6.5 expected for each), so they do not support the hypothesis that even a subset of ER alleles are cis-acting. In fact, considering that we were able to find strong evidence of cis hits among the GR QTL, this result suggests that nearly all ER QTL in our list are likely to be trans-acting.

Repeating the male/female overlap test on our lists of ER QTL, we found seven transcript/marker pairs shared between the two lists (Fig. 3d), which is only slightly more than the random expectation of 1.2. This is in contrast to the much larger overlap between male and female GR hits (Fig. 2d). This lack of male/female overlap is due to a marked sex-specificity of ER QTL (see Materials and Methods), quite unlike the non-sex-specific GR QTL.

We next tested predictions made by a subset of the ER QTL in an independent data set. We generated genome-wide gene expression data from the livers of seven female mice from each of two strains (A/J and C57BL/6J [B6]) that were part of our original 19 strain data set. Even though the micro-environmental variation in this new data set is completely independent from that in our original data set, we predicted that the ER QTL would have a similar buffering effect. In two sets of genes, where expression levels were predicted to be more variable in either A/J or B6, we observed confirmation rates of 61% (34/56 genes) and 63% (17/27 genes) respectively (see Materials and Methods), and the two distributions of within-strain variance ratios were significantly different from one another (t-test p = 0.003). Although these results do not confirm the genomic positions of ER QTL, they do confirm the genetic basis of ER for over 50 genes. The 11 genes with the strongest confirmations are listed in Table 1; as with the genes buffered by GR QTL, a wide range of functional categories are represented. The top replicated gene, Hsd3b5 (involved in metabolism of steroid hormones), has a replication significance of p = 1.3×10^−6^. Interestingly, this transcript was highlighted by a previous study of B6 mouse livers that showed it to be among the most sensitive in the entire genome to environmental perturbations such as a common pollutant (di[2-ethylhexyl] phthalate) or dietary restriction [24]. Our results are quite consistent with this, and further suggest that Hsd3b5 transcript levels may not be as sensitive to such treatments in strains with the alternate ER QTL allele, such as A/J. As expected if ER QTL are sex-specific, 79 male ER QTL (identified from the full 19-strain data set) showed no evidence of replication in the independent female expression data (t-test p = 0.27).

**Table 1:** The 11 ER QTL with most strongly confirmed predictions.

Gene symbol	% within-strain variance explained by ER QTL	QTL chr	QTL pos	Predicted more variable strain	B6∶A/J log2 variance ratio
Hsd3b5	58.3	15	46057296	B6	7.6
T	62.7	9	43663988	B6	4.3
BE655403	57.8	6	80679828	B6	3.0
Olfr125	84.2	1	1.72E+08	B6	3.1
1110065F06Rik	56.5	11	63036066	B6	3.2
Spon2	63.6	2	77180992	B6	2.5
Chrna4	59.5	5	8411367	B6	3.2
Slc25a25	69.3	6	53955400	A/J	−2.7
Aacs	78.4	1	27827338	A/J	−2.9
Il23r	53.9	6	6583041	A/J	−3.1
Sdf2l1	54.3	1	1.84E+08	A/J	−2.6

Genes with ER QTL from the 19-strain set whose genotypes differed between A/J and B6 strains were tested in an independent group of A/J and B6 female livers (see Materials and Methods). The 11 genes with the strongest confirmation (measured by magnitude of the B6∶A/J variance ratio in the replication data set) are shown.

To test if ER QTL are tissue-specific, we profiled gene expression in the hypothalami and kidney cortexes of a subset of the 14 female mice used above (11 hypothalami and nine kidneys). In both cases we found no significant difference in the expression variance ratios for genes predicted to be more variable in A/J vs. B6 livers (t-test p>0.18 for both). This lack of replication cannot be attributed to the smaller number of samples used for hypothalamus and kidney, because restricting the liver replication analysis to the same individuals did not appreciably affect the results. Therefore we conclude that the ER QTL we have observed are likely to be tissue-specific.

### Comparing Genetic and Environmental Robustness Loci

Having successfully mapped both GR and ER QTL loci, we quantified the overlap between them. Strikingly, we found no cases where both ER and GR of an expression trait mapped to the same marker, using the genome-wide significance cutoffs described above. However, these cutoffs may be too stringent. A more sensitive test would be to restrict our search for GR QTL within only the ER QTL transcript/marker pairs while relaxing our GR QTL cutoffs because of the much smaller number of tests being performed. We restricted our search for ER QTL to within the GR QTL hits in a similar fashion.

Even with these more sensitive tests, we found no more overlap between ER and GR QTL than expected by chance. When searching for ER QTL within the list of GR hits found in both males and females (involving 78 transcripts), we tested five significance cutoffs for defining an ER hit, spanning a wide range of strengths (see Materials and Methods). The cutoffs were applied to both male and female ER analyses, resulting in a total of ten tests. The minimum p-value (assessed by randomization) for enrichment of ER hits within the GR list across all tests was 0.06, indicating no more overlap than expected at any threshold. The reciprocal test, searching for GR QTL within the significant ER hits, also showed no significant overlap: again applying five cutoffs to both male and female lists, the minimum observed p-value for overlap enrichment was 0.14, in agreement with the reciprocal enrichment test. Our power calculations for each test (above) indicate that this lack of overlap is unlikely to be due to insufficient power of the tests: taking into account our power to detect ER QTL (see Materials and Methods), at least ∼14 of the 78 significant GR transcripts would be expected also to be found as ER QTL if ER and GR QTL co-localized (probability of observing zero overlaps <10^−6^), and even more ER hits would be expected also to be found as GR QTL. Finally, simulations demonstrated our ability to identify joint ER+GR QTL when they exist (see Materials and Methods).

### Mapping Genetic and Environmental Robustness in *Arabidopsis* and Yeast

While the lack of overlap between ER and GR QTL in our mouse gene expression data was clear, whether this finding would apply to other traits and species was not. Therefore we extended our analysis to previously published life-history and morphological trait data from *Arabidopsis*. While the number of traits examined in these studies is necessarily small when compared to gene expression studies, they can at least indicate whether the patterns are consistent with the findings from our mouse study. In addition, since the number of strains used is much larger than in our mouse data, individual robustness QTL can be identified with much greater confidence.

**Figure 4:**
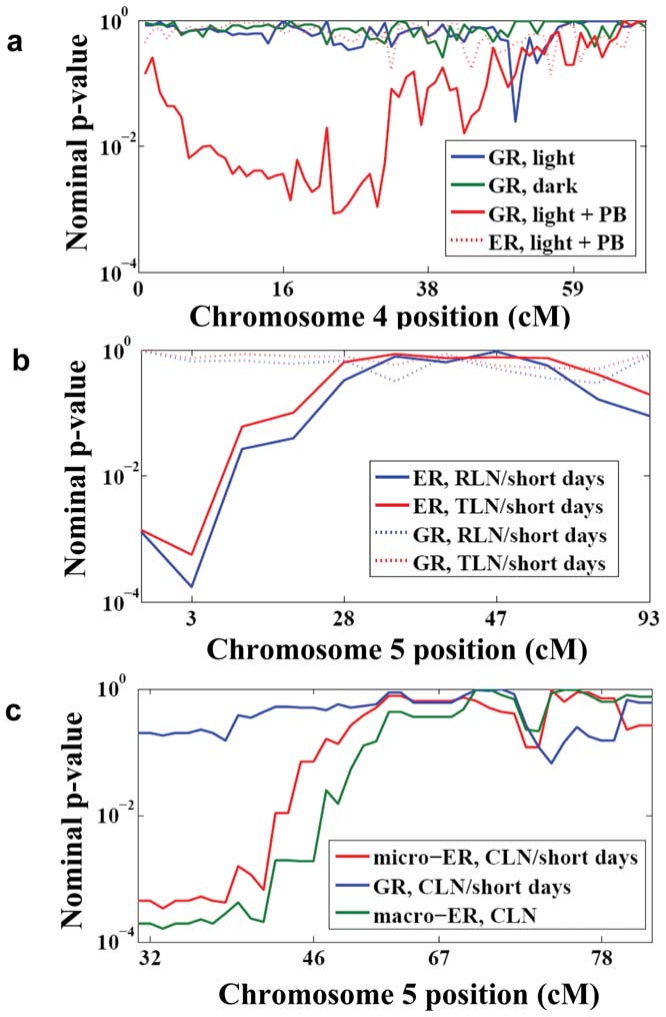
Genetic and environmental robustness QTLs in *Arabidopsis*. **a.** A GR QTL for seed germination timing [25] maps to chromosome 4. The QTL is specific to one environment (light + paclobutrazol [PB]), and there is no co-localized ER QTL. **b.** ER QTLs for two (highly correlated) traits, rosette leaf number (RLN) and total leaf number (TLN) [26], map to chromosome 5. There is no co-localized GR QTL. **c.** An ER QTL for cauline leaf number (CLN) [27] in short days (“micro-ER”) is located on chromosome 5. This co-localizes with a macro-ER QTL for CLN, but not with any GR QTL. The figure begins at ∼32 cM into chromosome 5 because to the left of this position are two QTL for trait means, and thus these regions are not considered for ER or GR QTL mapping.

In one published study [25], the germination time of seeds from a set of 98 recombinant inbred lines (RILs) was measured in three environments (light, dark, and light+the gibberellin inhibitor paclobutrazol [PB]). An average of ∼12 plants from each RIL were tested in each environment, which is sufficient replication to allow both ER and GR mapping within each environment. We found one significant GR QTL (Fig. 4a; uncorrected p = 0.001, genome-wide permutation across all three environments p = 0.039), but no ER QTL that even approached genome-wide significance. Interestingly, this GR QTL was found in only one of the three environments, suggesting that GR QTL can be condition-specific (see Materials and Methods). A sensitive test for ER QTL co-localization with this GR QTL is to test just the most significant GR QTL marker, since then no correction for genome-wide testing is required. But even this test was not significant (p = 0.58; 99% confidence interval [CI] = 0.23–0.99), supporting the distinction between GR and ER QTL.

We also analyzed data from a study [26] in which four correlated phenotypes (flowering time [FT], rosette leaf number [RLN], cauline leaf number [CLN], and total leaf number [TLN]) were measured in two photoperiod environments, long days and short days. An average of ∼12 plants for each of 96 RILs (Nd x Col) were measured in each environment. Here we found no GR QTL, but a number of ER QTL: six of the eight possible trait/environment combinations had an ER QTL (uncorrected p<0.0006 for each; overall genome-wide permutation p = 10^−5^), which mapped to two loci (Fig. 4b and Fig. S3). Testing just these six most significant trait/marker/environment combinations, we found no evidence for overlapping GR QTL (all six had uncorrected p>0.2 and 99% CI lower-bound p>0.05).

Another *Arabidopsis* data set we analyzed [27] measured the same four phenotypes, in three environments: short days, long days, and long days with a vernalization treatment. Approximately 10 plants for each of 162 RILs (Cvi x Ler) were measured in each environment. We found a result similar to above, with six of the 12 possible trait/environment combinations having at least one ER QTL (Fig. 4c; uncorrected p = 0.001 for each, overall genome-wide permutation p = 0.003), but no significant GR QTL. Testing only the most significant ER QTL trait/marker/environment combinations for GR QTL, we still did not find any evidence for more than a slight (∼0.5%) chance of one co-localizing GR QTL (see Materials and Methods).

With this data set it is also possible to separately map QTL buffering different types of environmental variation. We define micro-environmental variation as variation within a single treatment group (e.g. long days), and macro-environmental variation as the variation between treatment groups. Macro-environmental variation can be measured by taking the median trait value for each strain within each experimental condition (thereby removing micro-environmental fluctuations, as in the first step of GR QTL mapping) and then calculating the within-strain standard deviation for each trait median across all conditions tested. With only three macro-environments in the experiment [27], estimates of macro-environmental variation are based on only three data points per trait and thus may be subject to a great deal of error, but they will at least be independent of the micro-environmental variation (note that all the ER QTL we report in mice are micro-ER QTL). We found “macro-ER QTL” for all four traits, using the same significance cutoffs as for “micro-ER QTL” above. These occurred at two loci, one of which was in precisely the same location as a micro-ER QTL affecting CLN variation in short days (Fig. 4c). Although co-localization of QTL does not prove a single factor is responsible for both, the overlap is nevertheless striking, and is consistent with the hypothesis that the same polymorphism(s) are buffering very different types of environmental variation.

To determine the pattern of ER/GR QTL overlap in a species aside from multicellular diploids such as mouse and *Arabidopsis*, we also analyzed a set of morphological traits in haploid *S. cerevisiae*, a unicellular yeast. Using automated high-throughput microscopy, 281 traits were previously measured in 62 yeast strains from a genetic cross between two diverged parental strains [15]. Because these measurements were performed on over 600 different individuals of each strain from three replicate cultures, the within-strain variability in each trait is reflective of micro-environmental and stochastic differences; within-strain coefficients of variation (CVs) were reported for 220 traits. These CVs were compared to strain genotypes, and 25 CVs were found to map to 28 QTL where the trait means did not also map [15]. Genetic mapping of within-strain trait CVs is precisely equivalent to our ER mapping, so we used the 25 morphological traits with ER QTL as input for our GR mapping algorithm. At all significance cutoffs tested–including one lenient enough to allow the GR of all 25 traits to map somewhere in the genome–we found no overlaps between the locations of GR and ER QTL for any trait, consistent with the findings from mouse and *Arabidopsis*.

## Discussion

Our results suggest that many naturally occurring polymorphisms may buffer genetic variation, and that these polymorphisms are generally distinct from those buffering environmental variation. We found similar patterns for mouse gene expression levels, *Arabidopsis* life-history traits, and yeast morphological phenotypes, implying that this segregation of buffering effects is not limited to any one particular class of traits or species. Underscoring the lack of overlap between different classes of buffering QTL, for mouse gene expression traits we found differences in their mechanistic properties as well: ER QTL are mostly sex-specific and trans-acting, whereas GR QTL are not sex-specific and often cis-acting (the reason for this unexpected difference will be an interesting subject for future work). Unlike for GR/ER QTL, we found that macro-ER QTL do overlap micro-ER QTL, suggesting that the same polymorphism(s) might be able to buffer very different types of environmental variation. Both the independent replication of ER QTL and our finding of many cis-acting GR QTL support the validity of these preliminary results.

Our approach was designed to separate the effects of genetic and environmental variation by measuring both within- and between-strain phenotypic variation, but it remains possible that this separation was not perfect. For example, if some inbred lines were not precisely genetically identical then some genetic variation may have contributed to our within-strain variation, or if taking the median trait values for genetically identical individuals failed to “average out” all micro-environmental effects then some environmental differences may be a component of our between-strain variation. However, both of these effects (as well as any others that reduce our ability to separate sources of variation) will tend to *increase* the overlap between ER and GR QTL, and thus make our results conservative.

It should be noted that the within- and between-strain trait variation we have studied can have many potential sources. Aside from false positive associations that occur in any studying mapping thousands of traits (which we account for by permutation testing; see Materials and Methods), the only statement we can make about the sources of the phenotypic variation we observe is that they are ultimately rooted in the genetic differences between strains in our panel. We cannot make any inferences about the actual biological mechanisms by which these alleles exert their effects. For example, phenomena such as behavior could be involved: if one strain has a more variable sleep/wake cycle than another, the expression of many genes may show a concomitant increase in variability. Alternatively, genetically-rooted differences in the abundances of cell types in the livers of different strains could also give rise to ER or GR QTL. While these scenarios do technically fit the definition of robustness used throughout this work, whether robustness should be defined to exclude such cases is an issue we will not attempt to address here.

These findings have a number of implications for our understanding of canalization and evolution. Polymorphisms are the raw material for natural selection, so studying the properties of polymorphic alleles reveals what avenues are available for evolution. Of course we have only examined a snapshot of polymorphisms, and it is entirely possible–indeed, almost certain–that some polymorphisms are capable of buffering both genetic and environmental variation. If these dual buffering alleles are in fact exceedingly rare, as suggested by this analysis, then they would have to confer a vastly superior fitness advantage to be driven to fixation (100% frequency within a species) at higher rates than the far more common alleles buffering just one type of variation. Based on theoretical work [2]–[3] a great fitness difference seems unlikely (see Materials and Methods), so we favor the hypothesis that canalizing factors fixed in a species will also usually buffer only one type of variation. It is even possible that most canalizing factors are fixed by neutral drift, in which case the properties of common buffering polymorphisms (including their tendency to confer GR or ER, but not both) would closely parallel the properties of fixed alleles, because the fixed alleles would be a random subset of the common polymorphisms. Indeed, the fact that even traits important to fitness (such as germination time and flowering time in *Arabidopsis*) have robustness QTL suggests the absence of strong stabilizing selection and a more dominant role for neutral drift (see Materials and Methods for further discussion). However in the (perhaps rare) instances when buffering of a trait becomes strongly (dis)advantageous, our finding that abundant genetic variation in phenotypic robustness exists implies that selection could often act rapidly by fixing existing alleles.

The ability to genetically map both ER and GR QTL, along with the knowledge of the existence of GR QTL, opens up possibilities for future work. One important question that could be addressed is whether robustness QTL affect complex human disease phenotypes, in addition to the phenotypes studied here. Existing data from genome-wide association studies (GWAS) in humans could be re-analyzed to find polymorphisms affecting the within-genotype variation in clinically important quantitative traits; these would almost surely be missed by current approaches to analyzing GWAS, and may increase our understanding of the risk factors involved in complex diseases [28]. If robustness QTL are identified, twin studies could then reveal whether such polymorphisms affect ER or GR: ER QTL would have the property that monozygotic twins with the less robust genotype would show greater difference from one another than those with the more robust genotype (analogous to the greater within-strain variance of the less robust ER QTL allele), while GR QTL would show no such difference in twin variability.

Many other avenues for follow-up work exist as well. For example, recombinant inbred lines established from normally isolated races or interfertile species could reveal buffering effects of factors fixed since the divergence of those populations. Knowledge of the properties of canalizing polymorphisms will allow more empirically-grounded theoretical work on the evolution of canalization, as well as potential roles for robustness QTL in phenotypic plasticity and evolvability, to be carried out. Experiments involving different types of environmental variation (e.g. field studies) may reveal many new ER QTL, whose properties could be compared with the laboratory-based ER QTL reported here. And finally, we can now start to examine ER and GR QTL for signs of natural selection acting upon them. This may point us towards the answer to another fundamental question: whether canalization is most often a product of natural selection, or just a by-product of random drift.

## Materials and Methods

### Data Generation

The 19 inbred lines profiled for this study were 129S1/SvImJ, A/J, AKR/J, BALB/cByJ, C3H/HeJ, C57BL/6J, C57BLKS/J, C57L/J, CAST/EiJ, CZECHII/EiJ, DBA/2J, FVB/NJ, LG/J, NOD/LtJ, NZB/BlNJ, PERA/EiJ, SJL/J, SM/J, and SWR/J. All mice were reared at Jackson Laboratories in Bar Harbor, ME and shipped to Jackson Laboratories in Sacramento, CA (JAX West) at 7 weeks of age. Mice were maintained on a 12 h light-dark cycle and fed ad libitum (Purina Chow from Ralston-Purina Co., St. Louis, MO). At 20 weeks of age all mice were euthanized and liver tissues were collected, flash frozen in liquid nitrogen, and stored at −80 degrees C prior to RNA isolation. In addition, 14 females from two inbred lines (A/J and C57BL/6J) used for replication of ER QTL were reared at The Mouse Clinical Institute in Strasbourg, France. All mice were maintained on a 12 h light-dark cycle and fed a chow diet ad libitum until 7 weeks of age, at which point they were switched to a high-fat diet. At 16 weeks of age all mice were euthanized and liver, hypothalamus, and kidney cortex tissues were collected, flash frozen in liquid nitrogen, and stored at −80 degrees C prior to RNA isolation. All procedures of housing and treatment of animals were performed in accordance with IACUC regulations.

RNA preparation and array hybridizations were performed at Rosetta Inpharmatics. The custom ink-jet microarrays were manufactured by Agilent Technologies (Palo Alto, CA). A custom array was designed for this study and consisted of 39,280 non-control oligonuceotides extracted from the mouse Unigene clusters and combined with RefSeq sequences and RIKEN full-length cDNA clones. Mouse liver tissues were homogenized and total RNA extracted using Trizol reagent (Invitrogen, CA) according to manufacturer's protocol. Three µg of total RNA was reverse transcribed and labeled with either Cy3 or Cy5 fluorochrome. Labeled complementary RNA (cRNA) from each animal was hybridized against a cross-specific pool of labeled cRNAs constructed from equal aliquots of RNA from representative animals for each strain. The hybridizations were performed in fluor reversal for 24 hours in a hybridization chamber, washed, and scanned using a confocal laser scanner. Arrays were quantified on the basis of spot intensity relative to background, adjusted for experimental variation between arrays using average intensity over multiple channels, and fitted to a previously described error model [29] to determine significance (type I error). Alternative normalization, such as median-centering, did not appreciably affect our results. All microarray data are MIAME compliant and have been deposited in the NCBI GEO database under accession number XXXXX.

### ER and GR Mapping

For both ER and GR mapping in mouse, we only considered transcripts with σ>0.15 (median within-strain σ for ER mapping and between-strain σ for GR mapping), since probes with little or no variability across all samples are unlikely to show large differences in variability between subsets of samples. The results were largely robust to variation in this threshold, with the primary effect being that decreasing the stringency increased both the FDR and the number of hits. Any differences in transcript lists were taken into account when calculating overlaps between lists. In addition we restricted our analyses to the ∼9,000 most informative markers, namely those with allele frequencies closest to 50% (minor allele present in 9/19 strains), to maximize our power for genetic mapping.

For all analyses we excluded any QTL for trait medians, to focus on effects restricted to trait variability. We used the same correlation approach as for ER QTL (above) to find QTL for medians (with an uncorrected p<0.01 cutoff). Because these p-value cutoffs determined what trait/marker pairs we *excluded* from our analysis, not correcting for multiple tests is conservative; if we were to take into account the tens of millions of tests being performed in each QTL scan of the mouse gene expression data, the nominal p-value needed to pass a Bonferroni correction would be ∼10^−9^, meaning that far fewer trait/marker pairs with moderate-strength “typical” QTL would be excluded.

The basis for our GR mapping is the median variant of the Fligner-Killeen test [20]–[21]. In this test, all data from two or more median-centered groups are merged into a single list and then ranked. The difference between each rank and the median rank is then combined within groups and used as a measure of each group's dispersion:
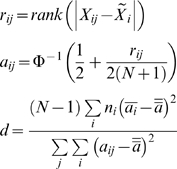
where *k* groups (in the present context, *k* = 2) are represented by index *i* and individual data points within each group by index *j*; *X_ij_* is a data point; *N* is the total number of data points in all groups; *n*_i_ is the number of data points in group *i*; Φ^−1^ is the inverse normal function; 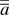 is the grand mean *a* value; 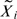 is the median *X_ij_* value for group *i*; and 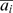 is the mean *a* value for group *i*. The test statistic *d* is distributed as a χ^2^ with *k−*1 degrees of freedom; a p-value computed based on *d* represents the probability of observing a particular GR QTL by chance (although we do mention these p-values in the text, we assess their true significance by permuation, described below). We note that when any *n*_i_ is odd, then one element of 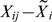 is guaranteed to be zero, which introduces a slight bias against identifying the odd-numbered group *i* as a high-dispersion subset if at least one other *n*_i_ is even. This bias shrinks with increasing *n*. One possible variant of the test that avoids the bias altogether is to replace 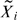 by 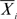; in practice this tends to increase the number of hits but also the FDR at a given significance threshold, so we have not used it in this work. Since the bias applies equally to our permuted data, it did not affect our statistics. Note that a maximally significant Fligner-Killeen test result for a given sample size does not imply that a particular marker's genotype explains all of the variance in the phenotype being mapped (as a maximally significant result would indicate in the context of traditional genetic mapping), but rather that the dispersions of the rank order of phenotype values for each genotype are as different as they possibly could be.

ER mapping was done by calculating a within-strain standard deviation for each trait, separately for males and females. A linear Pearson correlation (precisely equivalent to a t-test in this context) was then calculated between these standard deviations and the genotypes at every eligible genetic marker. In the mouse analysis the genotype had to explain at least 50% of the variation in within-strain variability (corresponding to a nominal p<0.00085) to be counted as an ER QTL (this does not mean that each gene can have only one significant marker, since marker genotypes can be highly correlated with one another, and so can explain overlapping portions of the variance). Correcting for the median or mean (by using the coefficient of variation) did not substantially impact our results, because all “typical” QTL—even extremely weak ones (nominal p = 0.01)—were already excluded from the analysis.

All p-values (except where noted as uncorrected p-values) were estimated for both GR and ER mapping by randomly permuting genotypes, so that one strain's genotypes were paired with another's phenotypes, and then comparing the observed results to the distribution of permuted results. This preserves the correlation structure both between markers and between traits, and thus is an appropriate method to estimate p-values. We note that in these inbred mouse lines, many unlinked markers show strong correlations with each other, which can lead to a single trait having apparent QTL at many different loci, when in fact only one is truly causal. While such effects will tend to dilute our signal and make it harder to pinpoint the causal locus, they will not cause the appearance of false QTL when none actually exist. Most importantly, any effects of marker genotype correlations will be accounted for by our permutation strategy.

These permutation tests allow us to estimate the false discovery rate (FDR) among GR and ER QTL, by comparing the observed number of QTL to that seen in the randomizations. For GR QTL, we found FDRs of 63.1% in males and 58.1% in females. For ER QTL, the FDRs are 72.0% for males and 70.1% for females. These high FDRs underscore the notion that with only 19 strains, we can only provide a coarse-grained view of these QTL. The fact that ER QTL can be confirmed (Table 1), and that GR QTL are often cis-acting, both support the notion that our QTL list contains many true-positives.

Combining the male and female GR QTL would have led to an overestimate of the number of significant loci in this intersected set if randomizations were done separately for males and females. This is because the male and female data are not actually independent (they share genotypes), so more overlap would be expected by chance than would be estimated from separately permuting each gender's data and then intersecting the results. For this reason, we permuted the male and female data together (e.g. the genotypes of strain X were paired with the phenotypes of strain Y, for both males and females), and estimated the expected overlap from these shared permutations.

To estimate the fraction of true-positive GR hits that is cis-acting, we calculated the overall number of true-positives (total hits minus false positives) involving microarray probes that could be assigned to a unique genomic location and the number of cis-hit true-positives (cis hits minus false positives). The ratio between these two is equal to the approximate fraction of true-positives that act in cis (this follows from the fact that cis-QTL are a subset of all QTL, we used the same thresholds for cis and trans-hits, and we have estimates for the number of true positives of each). For males this is 35%; for females, 34%. The analogous estimate for cis-acting ER hits yields zero for males (since the observed number is slightly below the expected), and 0.8% for females, which is statistically indistinguishable from zero.

We calculated enrichments of Gene Ontology annotations near significant GR QTL, but did not find any significant enrichments (though our power to detect any enrichments was low, due to the large size of genomic regions associated with most transcripts). One significant marker was located close (55 kb) to one of the three HSP90 genes in the genome, but this is not unexpected given the large number of significant markers. To perform this analysis we tested both the closest gene to each marker, and also all genes within 100 kb. As controls we used randomly chosen markers, as well as markers resulting from GR QTL permutation runs.

### Testing of ER QTL Predictions

Testing of the ER QTL in additional A/J and C57BL/6J mice was performed as follows. To be included in the set of predictions tested, a gene had to be the target of an ER QTL whose genotype differed between A/J and C57BL/6J, and also had to have no more than one missing data point in each of the two groups of replication microarrays. We defined confirmation as the correctly predicted strain having higher variation. Because the ER QTL make relative (not absolute) predictions about within-strain variation, we used the average within-strain variance ratio for genes not predicted to be different between the two strains as a baseline for comparison to our predictions (to account for the possibility that one strain tends to be more variable in general than the other, which would skew the confirmation results). With a sample size of seven mice of each strain, a variance ratio of 5.8 is needed to reach a nominal significance of p = 0.05 (by F-test). Among the 83 genes tested for replication in female liver, by chance we would expect to see ∼2.1 genes agreeing with our prediction of which is the more variable strain at this two-sided p = 0.05 cutoff, and ∼2.1 disagreeing. Consistent with the latter, we found 3 genes disagreeing; however we found 11 genes in agreement (Table 1), which is significantly (p = 10^−6^) greater than the 2.1 expected. The top gene, Hsd3b5, had a 194-fold (7.6 on a log_2_-scale) difference in variance between B6 and A/J, yielding an F-test p = 1.3e-6. Given that 83 genes were tested, the probability of observing one replicating this strongly by chance is 83×1.3e-6 = 0.0001.

### Testing for Confounding Effects

Because the mice in our experiment were kept in multiple cages and shipped to JAX West on multiple dates, we tested whether this may have introduced systematic errors (“batch effects”) into our data. We classified every pair of mice with the same strain and sex into three partially overlapping groups: those that shared a cage (*n* = 116), those that were shipped together (*n* = 94), and those that were neither caged nor shipped together (*n* = 359). If cages or shipping affected our data in any systematic way, we would expect expression levels from pairs in one or both of the first two categories to be more highly correlated than pairs in the third category. However, we observed similar average correlations in all three groups: *r* = 0.691, 0.706, and 0.685, respectively (σ>0.11 for all three). Therefore we conclude that cage and shipping effects did not greatly influence the expression data.

Our permutation method does not account for the population structure of the mouse strains. Because some strains are more closely related than others, this could in theory introduce artifactual QTL, for example if one monophyletic clade had more variability for a trait than another (the potential pitfalls of measuring canalization in groups with unequal genetic variation have been stressed previously, e.g. [10]). Under any type of population stratification, more closely related strains would co-occur in the same genotype class (e.g. the high-dispersion class in GR mapping) more than expected by chance (based on all tested markers). To test if such stratification influenced our results, for all 171 possible pairs of our 19 mouse strains we compared phylogenetic distance (measured by the number of differing SNP genotypes across all markers) to frequency of co-occurring in a genotype class for either ER or GR QTL. For both types of QTL we observed no enrichment of co-occurrence for closely related pairs, indicating a lack of population stratification in this data set. As a second test of population structure, we plotted the cumulative distribution of p-values for all four of our QTL scans (ER and GR QTL in males and females), as done previously by numerous studies (e.g. [30], as well as nearly all genome-wide association studies of human populations). Under the assumption that only a tiny fraction of marker/trait pairs will have true associations, the distribution should follow a straight line with slope = 1 in the absence of population structure (or any other confounding factor) inflating p-values in a systematic fashion. All four plots showed the absence of inflated p-values (Figure S4).

Another possibility not accounted for by permutation is if our GR QTL actually resulted from “regular” QTL for trait mean where one allele of the causal variant was present at a ∼50% frequency in the high-dispersion genotype class, but not present in the low-dispersion class. Such a case could result in an increase in dispersion for one genotype, which is the signature of GR QTL. However regardless of the strength of this hypothetical regular QTL and the causal allele frequencies in each genotype class, our simulations of this scenario showed that the effect on trait mean is detected much more easily than the effect on dispersion, so that the effect on trait mean must be very significant (p≪0.01) before having even any appreciable effect on the dispersion. Because we use a very conservative (for our purposes) cutoff of nominal p = 0.01 to disqualify any marker/trait pair showing evidence of a regular QTL from consideration as GR QTL, regular QTL will not be falsely detected as GR QTL.

To test if any of our results may be affected by the presence of SNPs within the microarray probes used to measure expression levels, we compiled a list of 1,428 probes overlapping known SNPs (3.6% of all probes). We found no significant enrichment for this subset of probes in any set of our GR or ER hits, when using either the entire genome or the subset of ∼2,000 most variable probes as the background set. This lack of enrichment indicates that hybridization artifacts due to SNPs within microarray probes are unlikely to affect our results.

Finally, one factor that may at first appear to be a possible confounder in the ER QTL analysis is behavioral differences between strains that could cause one strain to have a higher variance for some trait than another strain. However even though this may well occur, it is perfectly consistent with the ER QTL framework. To illustrate this in the setting of “typical” QTL, imagine a study that mapped typical QTL for obesity in mice. These QTL could cause obesity through changed behavior (e.g. eating more or exercising less), or alternatively through a different mechanism (e.g. changed metabolic rate). In either case, however, it is a genetic effect on obesity. The same holds true for ER QTL: even if they are due to changed behavior, this behavior is rooted in genetic differences between strains, which affects environmental robustness and leads to ER QTL.

### Testing Sex-Specificity and Power

To determine if the GR QTL were sex-specific, we compared the number of QTL found separately in males and females to the number found when the same set of 370 mice were split into random halves, irrespective of their genders. Each half had the same number of samples as in analyses separating mice by sex, but since the partitioning was random, the number of hits in each list could be used to determine if separating by sex is any different than random segregation. In over one thousand random partitionings of the data we observed an average of 5,140 (σ = 272) significant transcript/marker pairs, very close to the male/female average of 5,129, indicating that the GR QTL can be detected equally well in both single-and mixed-sex populations, and thus they are not sex-specific.

Power of the ER mapping was estimated by randomly partitioning the data into two groups (with the constraint that as close as possible to an equal number of mice from each strain were included in each group), performing ER mapping, and calculating four values: the FDRs for ER hits from each group (based on 100 genotype randomizations), the overlap between groups, and the overlap between false discoveries (from the same 100 randomizations). Because there are millions of possible marker/trait combinations, overlaps between truly random pairs would be negligible; but the false positives from each of our two randomly chosen groups are not “truly random” in this sense, because they share the same genotypes. For this reason we first used the randomizations to estimate the frequency of overlaps among false positives (8.2%), and then used this to calculate the overlaps among true positives as all overlaps minus the expected false positive overlaps. We then calculated power as the ratio of true positive overlaps to total true positives. This entire process was repeated over 1,000 times to ensure an accurate estimation of power. The resulting figure of 28.5% should be considered a lower bound because in the presence of sex-specific effects, the signal/noise ratio (and thus power) will be much higher in a population of only males or females than in a mixed population of the same size. Because our power calculations were done in mixed populations, it will be an underestimate of the power we have in each single-sex group. Although this test has less power than GR mapping, with this power we would still expect to see at least ∼165 transcript/marker pair overlaps between males and females in the absence of sex-specific effects, in contrast to the seven that were actually observed. Therefore we concluded that the lack of overlap between males and females was not due to a lack of power; instead, ER QTL in this data set are mostly sex-specific.

The expectation of ∼165 overlaps in the absence of sex-specific effects was calculated as follows: expected overlap = (number of significant transcript/marker pairs)*([fraction true positives]*[power]+[fraction false positives]*[overlap between false positives]). The number of significant GR transcripts expected also to be found as ER transcripts if ER and GR QTL co-localized was calculated as (number of GR transcripts)*(fraction true positives)*(power), which conservatively assumes no overlaps between false-positive ER and GR loci. For the analysis of ER and GR QTL overlap, the five cutoffs tested for ER hits within the GR QTL list were at correlation coefficients r = 0.6, 0.5, 0.4, 0.35, and 0.3 (explaining between 36% and 9% of the variance in within-strain variabilities). For the reciprocal test of GR hits within the ER QTL list, the five cutoffs were p = 7.5e-4, 1.5e-3, 3e-3, 6e-3, and 1.2e-2.

### Simulations

To test if the lack of GR/ER QTL overlap could possibly be due to our test having less power to detect ER QTL in the presence of GR QTL (or vice versa), we turned to simulations. We simulated data for 3×10^5^ traits, each of which had either a GR QTL, ER QTL, or GR+ER QTL. A measure of the relative power of our mapping methods is the ratio of true ER QTL detected as ER QTL (at some significance threshold) to true ER+GR QTL detected as ER QTL. If GR QTL have no effect on our ability to detect ER QTL, then this ratio should be close to one at all thresholds; if instead GR QTL weaken our power to detect ER QTL, then the ratio will be greater than one. Strikingly, at all 10 thresholds tested, the ratio was between 0.99 and 1.01 (Fig. S5a), indicating that GR QTL do not affect our ability to map ER QTL. The reciprocal test for the effect of ER QTL upon power to detect GR QTL yielded similar results (all 10 ratios were between 0.97 and 1.01; Fig. S5b). In sum, our test is able to detect dual ER+GR QTL when they exist.

Simulated data (described above) were generated to mimic our male mouse expression data. For each trait, 182 individuals from 19 strains (91 of each genotype) were simulated. All individuals from genotype 1 were assigned random trait values sampled from a normal distribution with mean zero and σ = 1. To simulate ER QTL, individuals with genotype 2 were assigned random trait values sampled from a normal distribution with mean zero and σ = 1.5. To simulate GR QTL, individuals with genotype 2 were assigned random trait values sampled from a normal distribution with mean = +/−2 (+2 for half the strains and −2 for the other half) and σ = 1. To simulate ER+GR QTL, individuals with genotype 2 were assigned random trait values sampled from a normal distribution with mean = +/−2 and σ = 1.5. As in all analyses above, traits where the median value correlated with genotype at p<0.01 were discarded. For ER mapping the ten cutoffs were between correlation coefficient r = 0.2 and 0.65 in increments of 0.05, and for GR mapping they were between χ^2^ values of 2 and 11 in increments of 1. At each cutoff a ratio of ER (or GR) hits among true ER (or GR) traits to ER (or GR) hits among ER+GR traits was calculated. Altering the strengths of the simulated ER and GR QTL did not substantially affect these ratios.

### *Arabidopsis* and Yeast Analysis

*Arabidopsis* data were analyzed as follows. Genetic markers with >50% missing values were discarded, and plants heterozygous at any given marker (due to incomplete inbreeding) or missing a marker genotype were ignored in the analysis of that marker (neither of these filters excluded more than 1% of the data). The same nominal p = 0.01 cutoff as above was used to discard any marker/trait pairs where the marker was associated with trait median. Data permutations were conducted to estimate the probability that a given number of (GR or ER) QTL would be found with randomized genotypes. Bootstrapping was done by sampling the data points for each trait/environment combination with replacement, to generate bootstrap data sets that were then analyzed in the same way as the real data. All randomization p-values and bootstrap confidence intervals were based on at least 1,000 permutations/bootstraps. Several *Arabidopsis* data sets [31]–[34] were analyzed that did not contain any significant GR or ER QTL. For yeast, the median value for each trait across all three replicate cultures was used as input for the GR mapping, applied as described above.

In our analysis of the data from [25], we found that GR QTL can be condition-specific. The GR QTL from this data set (shown in Fig. 4a) was only seen in the environment with light+PB; at the most significant marker, uncorrected p = 0.43 in light and 0.72 in dark; Fig. 4a). Bootstrap testing (see Materials and Methods) revealed that at the most significant marker, the 99% CI for the p-value of GR in light+PB did not overlap with either the 99% CI for GR in dark or the 95% CI for GR in light, demonstrating the condition-specificity of this GR QTL. In our analysis of the data from [27], we found only a slight (∼0.5%) chance of one co-localizing ER/GR QTL. All six ER QTL from this data set had uncorrected p>0.02 for GR QTL, which is not significant after correcting for six tests; five of the six 99% CI lower bounds had p>0.01, with the sixth lower bound at p = 0.003, indicating a 0.5% chance that one of the six overlapped a GR QTL significant at the nominal p = 0.003 level.

### Expansion of Points Made in the Main Text

In the Introduction we state that “no loci influencing GR have yet been mapped”. While many examples of QTL for naturally occurring epistatic interactions that reveal cryptic variation exist, these have not been shown to affect GR. Alleles affecting GR must increase the variability of a trait across different genetic backgrounds, without affect the mean value (which is required since loci affecting the mean will almost always affect the variance as well).

In the discussion we state that it is unlikely a great fitness difference exists between polymorphisms buffering one type of variation (genetic or environmental) vs. both types. Let us imagine the case of stabilizing selection on a trait, i.e. selection for robustness. Theoretical work has shown that GR will be selected for only in a very narrow range of stabilizing selection pressures; and within this restricted range, selection for ER will be relatively weak compared to a higher level of stabilizing selection (for an illustration of this see Figure 9 of [3]). Therefore at no point in the spectrum of stabilizing selection strength is the fitness advantage of a dual ER+GR polymorphism very much greater than both ER and GR alleles alone (in most cases because the ER allele fitness effect dominates). We use this to reason that since single-type buffers dominate the common polymorphisms, they are also likely to dominate the fixed alleles, regardless of whether most fixation takes place due to selection or random drift.

Another point about the fitness effects of buffering polymorphisms is that it is entirely possible that dual buffering polymorphisms arise frequently but are strongly deleterious and therefore not present in our panel of strains due to negative selection. However, such deleterious variants are not relevant to questions of what possibilities are available for the evolution of robustness, because they will essentially never be driven to fixation if they are subject to strong negative selection. By focusing on natural variation, both for ER/GR polymorphisms themselves and for the polymorphisms being buffered by GR QTL, we are examining only what is most relevant to the evolution of robustness in natural populations. We note that while studies using genetic variation produced in the lab (such as complete gene deletion strains) can be quite informative in terms of the factors and mechanisms underlying robustness (e.g. [14]), they are less informative with regard to the evolution of robustness, since most gene deletions (or equivalent null alleles) are unlikely to become fixed—or even reach appreciable allele frequency—in natural populations. Nevertheless, if dual ER/GR buffers exist but have roles essential for life, then they would be invisible to our approach, since any variants affecting their buffering would be lethal (and the same drawback applies to studies of gene deletion strains, which are restricted to nonessential genes).

In the discussion we also state that “the fact that even traits important to fitness (such as germination time and flowering time in *Arabidopsis*) have robustness QTL suggests the absence of strong stabilizing selection and a more dominant role for neutral drift.” Although we find this hypothesis to be the most likely, some alternative explanations for having many robustness QTL do exist. For example a polymorphism underlying a robustness QTL could be under balancing selection, or could be currently undergoing a selective sweep; different alleles could have been fixed in different (normally isolated) populations that were each advantageous to their own environments; or the robustness QTL observed in laboratory settings might not be expressed as such in the wild. Finally, we note that the existence of robustness QTL also suggests the absence of strong destabilizing selection, i.e. selection for trait variability, which could be advantageous for bet-hedging strategies.

## Supporting Information

Figure S1 a. GR QTL in females. Transcripts are arranged in the genomic order of their genes along the Y-axis and genetic markers are in genomic order along the X-axis. Small black dots located at the intersection of a particular row and column indicate trans-acting hits between whatever trait/marker combination is represented by that row/column; larger red dots indicate cis-acting hits. Only genetic markers and traits with at least one significant hit in males or females are shown, and redundant markers (with identical genotypes) are included. b. GR QTL found in both males and females, with the same traits and markers as above.
(0.16 MB TIF)

Figure S2 a. ER QTL in females. Transcripts are arranged in the genomic order of their genes along the Y-axis and genetic markers are in genomic order along the X-axis. Small black dots located at the intersection of a particular row and column indicate trans-acting hits between whatever trait/marker combination is represented by that row/column; larger red dots indicate cis-acting hits. Only genetic markers and traits with at least one significant hit in males or females are shown, and redundant markers (with identical genotypes) are included. b. ER QTL found in both males and females, with the same traits and markers as above.
(0.09 MB TIF)

Figure S3 ER QTL for three (highly correlated) *Arabidopsis* leaf traits in long days, and for one leaf trait in short days, map to chromosome 2. There is no co-localized GR QTL.
(0.11 MB TIF)

Figure S4 Test of population structure in ER and GR QTL results. For each set of p-values, the cumulative distribution is shown (blue). The deviation from the diagonal line (red) is indicative of p-value inflation due to population structure or some other systematic bias, assuming that true marker/trait associations are extremely rare. For comparison see Figure 2 in Kang et al (2008). a. Male GR QTL p-values. b. Female GR QTL p-values. c. Male ER QTL p-values. d. Female ER QTL p-values.
(0.10 MB TIF)

Figure S5 a. The ratio of true simulated ER QTL detected as ER QTL (at a range of thresholds) to true ER+GR QTL detected as ER QTL. If GR QTL have no effect on our ability to detect ER QTL, then this ratio should be close to one at all thresholds; if instead GR QTL weaken our power to detect ER QTL, then the ratio will be greater than one. b. The ratio of true simulated GR QTL detected as GR QTL (at a range of thresholds) to true ER+GR QTL detected as GR QTL. If ER QTL have no effect on our ability to detect GR QTL, then this ratio should be close to one at all thresholds; if instead ER QTL weaken our power to detect GR QTL, then the ratio will be greater than one.
(0.10 MB TIF)
