# Supplementary figures and images for: The Quantitative Genetics of Phenotypic Robustness
Source: PLoS One. 2010 Jan 8;5(1):e8635. doi: 10.1371/journal.pone.0008635 (PMC2799522; doi:10.1371/journal.pone.0008635)

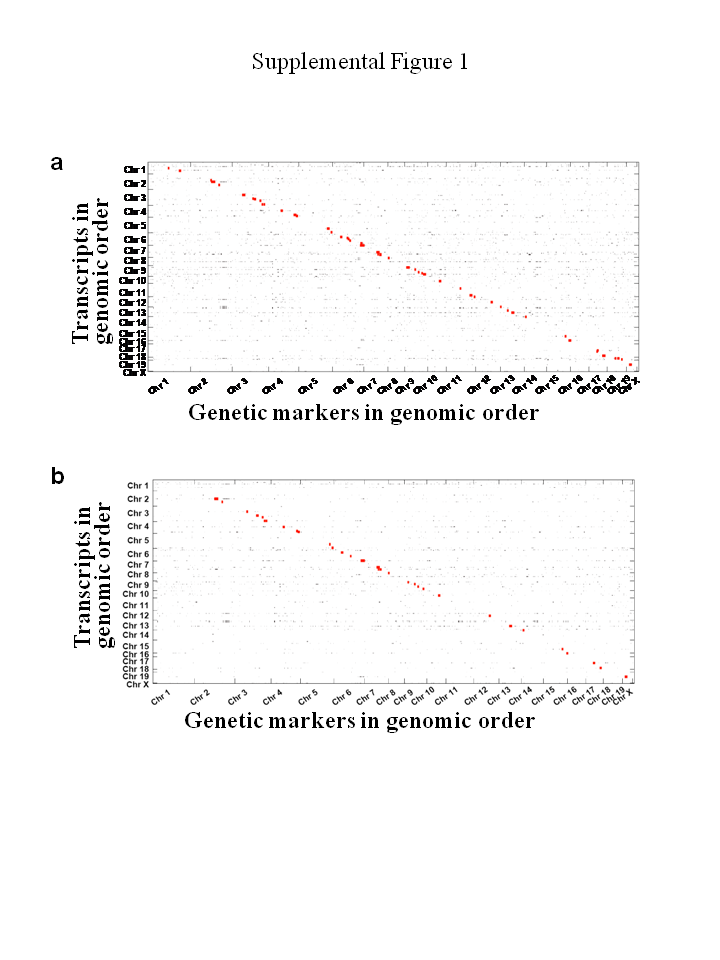

Supplement: Figure S1 — a. GR QTL in females. Transcripts are arranged in the genomic order of their genes along the Y-axis and genetic markers are in genomic order along the X-axis. Small black dots located at the intersection of a particular row and column indicate trans-acting hits between whatever trait/marker combination is represented by that row/column; larger red dots indicate cis-acting hits. Only genetic markers and traits with at least one significant hit in males or females are shown, and redundant markers (with identical genotypes) are included. b. GR QTL found in both males and females, with the same traits and markers as above. (0.16 MB TIF) [file pone.0008635.s001.tif]

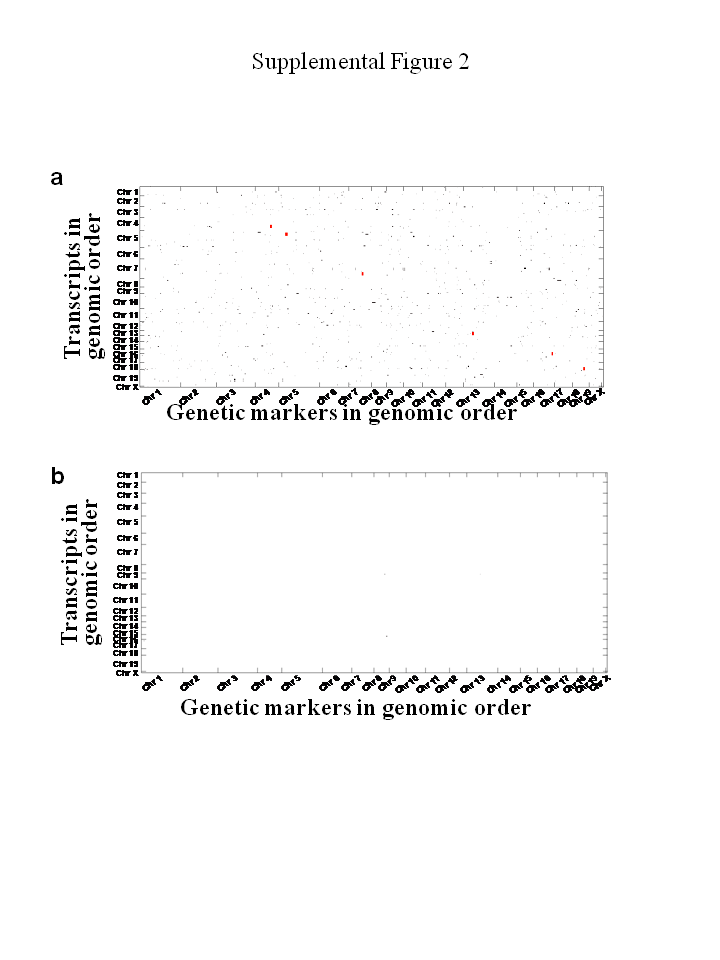

Supplement: Figure S2 — a. ER QTL in females. Transcripts are arranged in the genomic order of their genes along the Y-axis and genetic markers are in genomic order along the X-axis. Small black dots located at the intersection of a particular row and column indicate trans-acting hits between whatever trait/marker combination is represented by that row/column; larger red dots indicate cis-acting hits. Only genetic markers and traits with at least one significant hit in males or females are shown, and redundant markers (with identical genotypes) are included. b. ER QTL found in both males and females, with the same traits and markers as above. (0.09 MB TIF) [file pone.0008635.s002.tif]

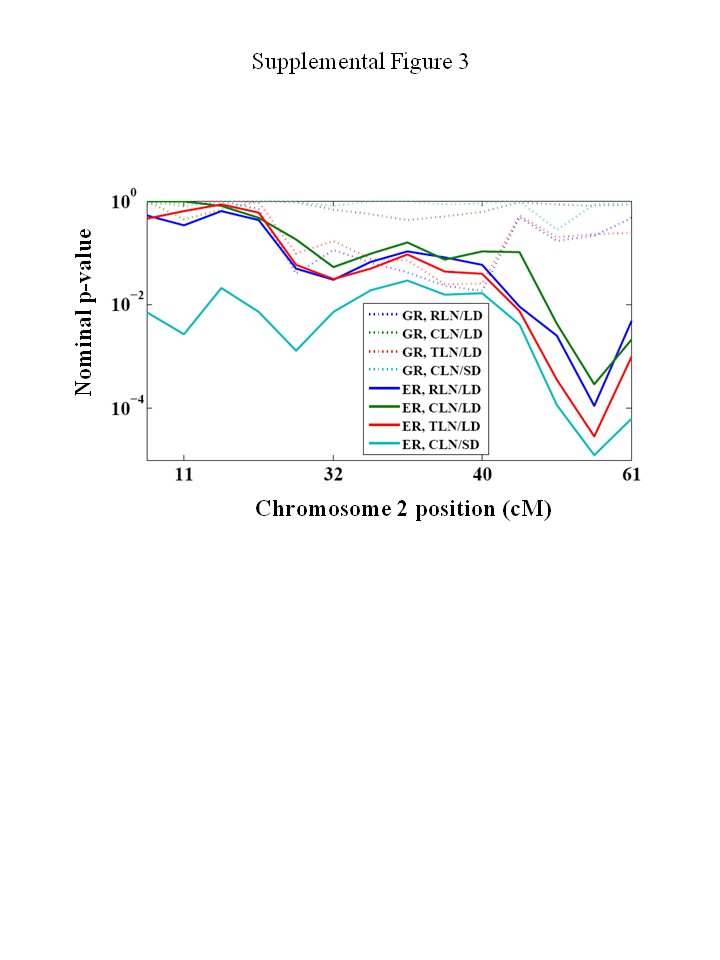

Supplement: Figure S3 — ER QTL for three (highly correlated) Arabidopsis leaf traits in long days, and for one leaf trait in short days, map to chromosome 2. There is no co-localized GR QTL. (0.11 MB TIF) [file pone.0008635.s003.tif]

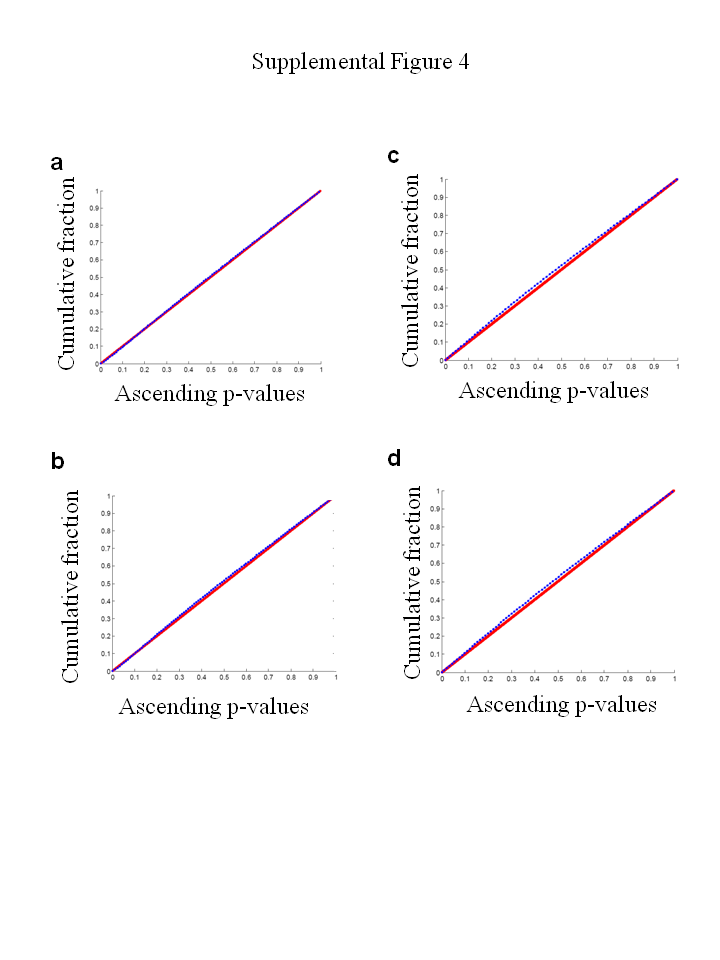

Supplement: Figure S4 — Test of population structure in ER and GR QTL results. For each set of p-values, the cumulative distribution is shown (blue). The deviation from the diagonal line (red) is indicative of p-value inflation due to population structure or some other systematic bias, assuming that true marker/trait associations are extremely rare. For comparison see Figure 2 in Kang et al (2008). a. Male GR QTL p-values. b. Female GR QTL p-values. c. Male ER QTL p-values. d. Female ER QTL p-values. (0.10 MB TIF) [file pone.0008635.s004.tif]

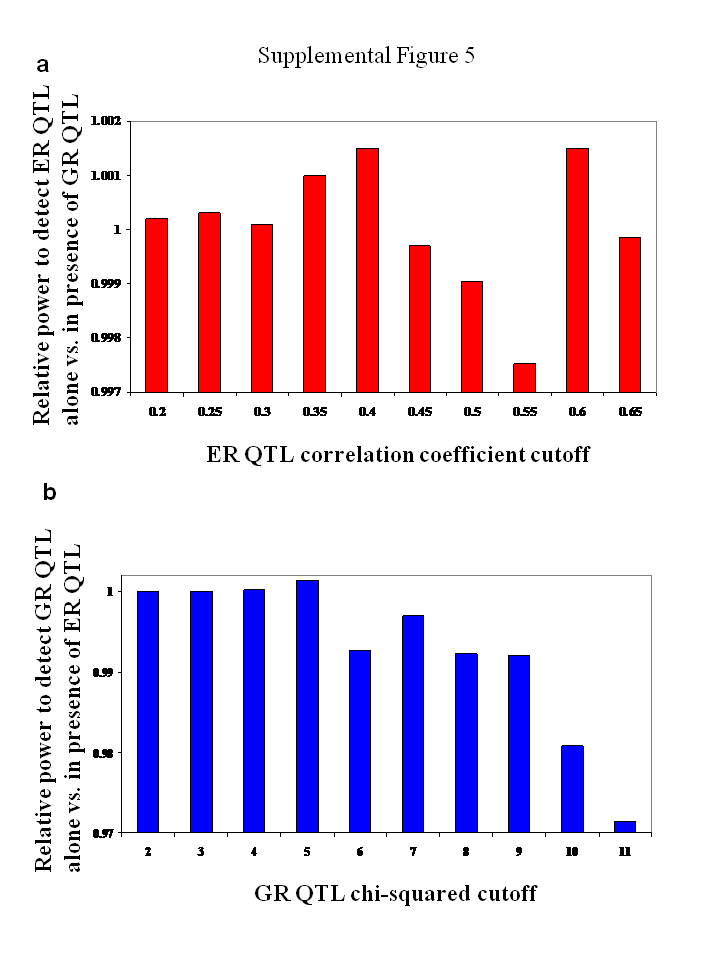

Supplement: Figure S5 — a. The ratio of true simulated ER QTL detected as ER QTL (at a range of thresholds) to true ER+GR QTL detected as ER QTL. If GR QTL have no effect on our ability to detect ER QTL, then this ratio should be close to one at all thresholds; if instead GR QTL weaken our power to detect ER QTL, then the ratio will be greater than one. b. The ratio of true simulated GR QTL detected as GR QTL (at a range of thresholds) to true ER+GR QTL detected as GR QTL. If ER QTL have no effect on our ability to detect GR QTL, then this ratio should be close to one at all thresholds; if instead ER QTL weaken our power to detect GR QTL, then the ratio will be greater than one. (0.10 MB TIF) [file pone.0008635.s005.tif]
